# Stress effects on framed decisions: there are differences for gains and losses

**DOI:** 10.3389/fnbeh.2013.00142

**Published:** 2013-10-09

**Authors:** Stephan Pabst, Matthias Brand, Oliver T. Wolf

**Affiliations:** ^1^Department of Cognitive Psychology, Institute of Cognitive Neuroscience, Ruhr-University BochumBochum, Germany; ^2^General Psychology: Cognition, University of Duisburg-EssenDuisburg, Germany; ^3^Erwin L. Hahn Institute for Magnetic Resonance ImagingEssen, Germany

**Keywords:** stress, cortisol, framing effect, gain/loss, decision making, risk, negative affect

## Abstract

Recent studies have shown that acute stress can lead to riskier decision making. Yet, the underlying mechanisms of the stress effects on decisions under risk remain poorly understood. To gain a better understanding of decision-making processes and potential strategy application under stress, we investigated decision making in pure gain and loss domains with unequal expected values (EVs) across alternatives. We conducted an experimental study with a 2 × 2 design (stress vs. no stress and gain domain vs. loss domain). The Trier Social Stress Test (TSST) was utilized to induce acute stress. Controls performed the placebo-TSST (p-TSST). To validate the stress response we measured salivary cortisol and alpha-amylase concentrations. We used a modified version of the Game of Dice Task (GDT) to assess decision-making performance in a gain and a loss domain. Results showed that non-stressed participants made less risky decisions in the gain domain compared to those of the loss domain. This behavior is in accordance with previous studies and indicates the stability of the framing effect in even more complex tasks with changing EVs across alternatives. Stress did not alter risk taking behavior in the gain domain. Yet, in the loss domain stressed participants made less risky decisions compared to controls. Additionally, the data support earlier findings of longer reaction times in loss compared to gain domains due to higher cognitive effort for loss-framed decisions. It is discussed that stress may lead to reduced amygdala activation, which has been found to reduce riskier decisions in a loss domain. With respect to earlier results of riskier decisions in tasks that unite both gain and loss domains, it is discussed whether stress leads to a stronger evaluation of high gains and a neglect of losses.

## Introduction

Decisions under risk are frequently made in a context of explicit and stable rules with foreseeable consequences of the specific alternatives (Brand et al., 2006). One way to experimentally measure performance of decisions under risk is the Game of Dice Task (GDT), a computerized game of chance (Brand et al., 2005). Riskier, i.e., poorer decisions in the GDT have been associated with lower executive functioning (Brand et al., 2007, 2009; Schiebener et al., 2011) and dysfunctions of the dopaminergic system as shown in patients with Parkinson's disease (Brand et al., 2004; Euteneuer et al., 2009; Labudda et al., 2010). Interestingly, in a series of studies we also observed riskier decision making after the induction of acute psychosocial stress and its associated increase in cortisol concentrations (Starcke et al., 2008; Pabst et al., 2013a,b). As the prefrontal cortex (PFC) is associated with executive functioning and feedback processing (Bechara et al., 2000; Fuster, 2000), both essential for advantageous decision making in conditions under risk (Manes et al., 2002; Brand et al., 2006), we interpreted riskier decision making as evidence for the negative effects of the stress hormone cortisol on PFC-mediated control functions. Support of our posit is found in stress-altered PFC activity (Pruessner et al., 2008, 2010; Dedovic et al., 2009b) and the influence of stress on dopamine efflux in the PFC (Butts et al., 2011).

In all trials of the original version of the GDT a die is thrown and participants are to guess what number will be shown. If participants are right money is won. Otherwise money is lost (Brand et al., 2005). Thus, in the original GDT participants are confronted with potential gains and losses. Research on the framing effect conducted within the framework of dual-process theories (Epstein et al., 1996; Kahneman, 2003) has revealed differences between decision making in gain and loss domains. Typically, in the gain domain risk avoidant behavior is shown by choosing a high winning probability associated with smaller gains. Contrary, in the loss domain risk seeking has been observed, manifested by choosing alternatives with a low loss frequency, but associated with high losses. Alternatives with more certain, yet lower losses are less likely to be chosen (Tversky and Kahneman, 1981). Such behavior has been found even when the economical outcome, known as expected value (EV; product of the probability-weighted value of a specific alternative), was worse for the more advantageous probabilities (high winning, low losing) compared to alternatives with the less advantageous probabilities (low winning, high losing; Aite et al., 2012; Koop and Johnson, 2012). This phenomenon has been interpreted as evoked by an emotional response bias reflecting a strong desire for gains and a strong aversion for losses (Camerer, 2005; Druckman and McDermott, 2008; Rick, 2011). Previous research has illustrated that the framing effect is susceptible to current emotional states of the decision maker (Williams et al., 2003; Cassotti et al., 2012). In addition, a first stress study has reported that stress, induced by the cold pressor test, leads to an enhancement of framing in both, gain and loss domains (Porcelli and Delgado, 2009).

As the GDT is a rather complex decision-making task, in the current study the attempt was to gain a better understanding of strategy use under stress in mere gain and loss domains. Hence, we modified the GDT presenting either a mere gain or loss domain. Further, we adjusted the monetary amounts of gains and losses to ensure equal increases of EVs with increasing winning and losing probabilities in each domain. Therefore, the least risky alternatives were most economical, also. As framed decisions are influenced by emotional biases (Tversky and Kahneman, 1981, 1986; Kahneman and Frederick, 2007) and EVs do not seem to be considered (Aite et al., 2012; Koop and Johnson, 2012), for non-stressed participants we expect no effect of altered EVs. Thus, among controls we expected the typical framing effect of less risky decisions in the gain and more risky decisions in the loss domain. Based on the results of Porcelli and Delgado (2009) stress should lead to an enhanced framing effect, with even less risky behavior in gain and riskier behavior in loss domains. Yet, pharmacological studies illustrate that high cortisol concentrations can lead to risky decision making, if the potential reward is high (Putman et al., 2010). Also, patients with amygdala lesions showed decreased framing effects in the loss domain (de Martino et al., 2010). Stress is thought to alter amygdala activity with a potential decrease (Pruessner et al., 2008; Merz et al., 2010) and may thus lead to a diminished framing effect in the loss domain. As the methodology of Porcelli and Delgado (2009) differs from ours (time of decision-making performance right after stress induction at a time where no cortisol increase is to be expected), we hypothesized that stress would lead to a diminished framing effect, which might be especially pronounced in the loss domain, i.e., less risky decisions in the loss domain.

An additional goal was to replicate earlier findings of longer reaction times in loss domains due to a higher cost-benefit tradeoff associated with higher cognitive effort in loss compared to gain domains (Gonzalez et al., 2005). Previous research by our group indicates that stress may not affect speed of GDT performance in the original version (Pabst et al., 2013b).

## Materials and methods

### Participants

Eighty (40 female) healthy students were recruited, aged 18–35 years, *M* = 24.29, *SD* = 3.54. They were randomly assigned to the four experimental groups: no stress—gain domain, no stress—loss domain, stress—gain domain, and stress—loss domain. We conducted a standardized telephone interview prior to the testing appointments, to ensure participants met the following requirements: no history of neurological or psychiatric disease, no smoking or drug abuse, no obesity (Body Mass Index [BMI in kg/m^2^] > 30), and no shift work. Also, anyone familiar with the stressor and the decision-making task was excluded. Women were not tested during menses and did not use hormonal contraceptives of any kind (Kirschbaum et al., 1999). Testing was conducted between 10:00 a.m. and 12:00. Participants were asked not to exercise and to abstain from alcohol 24 h and to get up 2 h before testing. Further, they were instructed not to eat and to merely drink water 1 h before testing. Written informed consent was given and €12 were received for participation. The Ethic committee of the German Psychological Association (DGPs) approved the study.

### Stress induction

We used the Trier Social Stress Test (TSST; Kirschbaum et al., 1993) for induction of acute stress. The TSST is an effective method to reliably activate the hypothalamic-pituitary-adrenal (HPA) axis (Dickerson and Kemeny, 2004), causing elevated cortisol concentrations. It also increases the activity of the sympathetic nervous system (SNS), as illustrated by increases in the enzyme alpha-amylase (Nater and Rohleder, 2009; Rohleder and Nater, 2009). Standing in front of a reserved and distant acting committee of two individuals (a man and a woman), participants had to perform two consecutive tasks. After a preparation time, a free speech had to be given, followed by a mental arithmetic (5 min each; total length of 15 min). In addition, participants were told that they were video-taped and that the video material would be used for further analyses after the task. Participants of the control group performed the placebo-TSST (p-TSST; Het et al., 2009), a non-stressful situation following the pattern of the TSST, but lacking all the socio-evaluative components. Participants were alone in the room giving a speech and performed a simplified arithmetical task.

### Measurement of affect

We administered the Positive and Negative Affect Schedule (PANAS; Watson et al., 1988) to assess positive and negative affect right before and after stress induction. Participants were to state how they felt at the very moment, answering 10 items for negative and positive affect, respectively, on a scale from 1 (*very slightly or not at all*) to 5 (*extremely*). We computed two scores using the total of either all positive or all negative items. A higher score indicated higher positive or negative affect, respectively.

### Assessment of biological stress markers

Salivary samples were collected by using salivette collection devices (Sarstedt, Nuembrecht, Germany) to assess alterations in endocrine activity at four points in time (1 min before and 1, 10, and 20 min after the TSST or p-TSST). Salivary cortisol concentrations served as an indicator of HPA axis activity (Dickerson and Kemeny, 2004). As an indirect marker of noradrenergic activity, we assessed salivary alpha-amylase (sAA; Nater and Rohleder, 2009; Rohleder and Nater, 2009). Samples were analyzed at the Laboratory of Professor Kirschbaum, Department of Biopsychology, Technical University Dresden, Germany. For free cortisol analysis a commercially available immunoassay was used (IBL, Hamburg, Germany). A quantitative enzyme-kinetic method was used for sAA analysis, as described somewhere else (Rohleder and Nater, 2009). Inter- and intra-assay variations were below 12%.

### Decision-making performance

To assess decision making in a gain and a loss domain, we modified the original GDT (Brand et al., 2005) with the result of a pure gain and a pure loss domain and the independent variable of domain as a between subject factor. The GDT is a computerized game of chance simulating decision making under risk. The goal in the gain and loss domains is to respectively maximize gains or minimize losses. Participants of the gain domain started with a capital of €0 and of the loss domain with €1000. In both domains, a single die is thrown in each of 18 trials. Beforehand, participants must choose from given and always present alternatives, consisting of a single number or a combination of two, three, or four numbers and thereby guess what number might be thrown. In the gain domain money is won, if the thrown number matches a single number or a number among the chosen combination. Else, no money is won. In the loss domain money is lost, if the thrown number does not match a number among the chosen alternative. Else, money will not be lost. Thus, in both conditions a match of numbers was perceived as positive. Feedback is given about the outcome and the current capital, after each trial.

**Figure 1:**
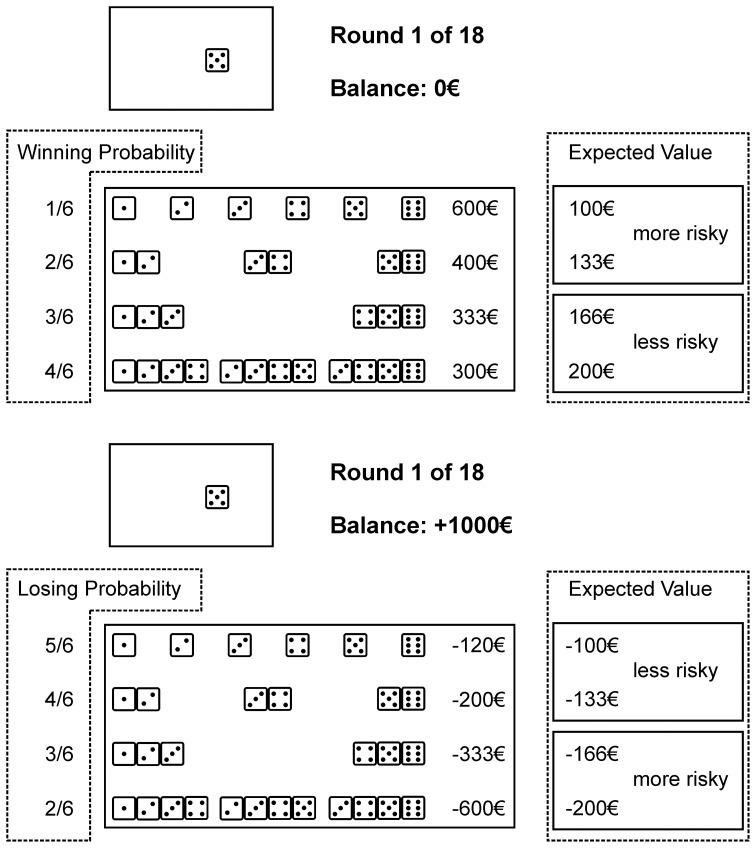
**A modified version of the Game of Dice Task, to measure decision making in situations of risk in a gain (above) or a loss (below) domain.** Participants follow the goal to maximize gains or to minimize losses, respectively. In the gain domain risk is defined by lower winning probabilities, higher gains, and concomitant lower expected values (single numbers and two number combinations). In the loss domain risk is defined by lower losing probabilities, higher losses, and lower expected values (three and four number combinations). Performance is measured by a net score computed by subtraction of the two more risky alternatives form the two less risky alternatives. For a detailed description of the task we refer to the method section (decision-making performance). Winning/losing probabilities and expected values were not displayed during task performance (information not provided for performance is highlighted by dashed lines).

With every combination a specific gain or loss is associated and a winning/not winning or losing/not losing probability. We classified the alternatives into more advantageous, i.e., less risky, and more disadvantageous, i.e., more risky, by the EV (i.e., the monetary gain/loss times the associated probability); a higher EV being more advantageous. For both domains higher winning/losing probabilities were associated with higher EVs. The amounts of the original GDT were adopted accordingly. In the gain domain, by choosing a single number €600 may be gained (winning probability 1 out of 6; EV €100). A combination of two numbers is associated with €400 (winning probability 2 out of 6; EV €133), a combination of three numbers may gain €333 (winning probability 3 out of 6; EV €166), and a four-number combination is associated with a €300 gain (winning probability 4 out of 6; EV €200). In the loss domain, by choosing a single number €120 may be lost (losing probability 5 out of 6; EV € −100). With a two-number combination €200 may be lost (losing probability 4 out of 6; EV € −133). A combination of three numbers may lose €333 (losing probability 3 out of 6; EV € −166), and a combination of four numbers is associated with a €600 loss (losing probability 2 out of 6; EV € −200). We refer to Figure 1 for graphical illustration.

Trial sequences of the GDT were: first, choosing one out of altogether 14 alternatives, as grouped into the mentioned four categories; second, the die is thrown; and third, feedback is given about any gain or loss, the current capital and the number of trials left. For performance measurement we computed a net score by subtraction of the more disadvantageous alternatives from the more advantageous alternatives. According to the earlier computed EV in the gain domain the more advantageous alternatives consisted of the three and four number combinations and the more disadvantageous alternatives of the single numbers and two number combinations. In the loss domain the more advantageous alternatives consisted of the single numbers and two number combinations and the more disadvantageous alternatives of the three and four number combinations. A higher net score indicated more advantageous decision making.

### Design and procedure

First, participants answered a demographic questionnaire followed by the PANAS and the first salivary sample at baseline right before the TSST or p-TSST, respectively. After the TSST or p-TSST the PANAS was administered again and the second salivary sample was taken. At approximately cortisol peak, 10 min after the TSST or p-TSST, the third salivary sample was taken and the GDT gain or the GDT loss was performed. Thus, participants were equally assigned to the four experimental groups of control gain, control loss, stress gain, or stress loss. Participants collected a fourth salivary sample after task completion, about 20 min after the TSST or p-TSST.

### Statistical analysis

We used SPSS 20.0 to conduct all statistical analyses. Groups were compared for age and BMI using a One-Way analysis of variance (ANOVA). Group differences for sex were analyzed using Pearson's χ^2^ test. Cortisol and sAA alterations and changes in positive and negative affect were detected by using an ANOVA with repeated measurement. We conducted a *t*-test for independent samples for sAA increase comparisons. A multivariate ANOVA was computed for decision-making performance and reaction time in the domains. For group comparisons concerning cortisol and sAA concentrations at different points in time of measurement and as follow up tests of group differences in decision-making performance and reaction time, simple effects tests were conducted. Two-tailed tests were performed with *p* set to 0.05 for all analyses.

## Results

Conducting a One-Way ANOVA we found no differences between the four groups for age (in years) or BMI (in kg/m^2^). Also, males and females were equally distributed between groups. Demographic values can be found in Table 1.

**Table 1:** **Demographic variables of the four experimental groups (each group *N* = 20)**.

	**Control gain *M* (*SD*)**	**Control loss *M* (*SD*)**	**Stress gain *M* (*SD*)**	**Stress loss *M* (*SD*)**	***F***	**χ^2^**	***df***	***p***
Age	24.60 (3.84)	24.65 (2.96)	23.10 (4.40)	24.80 (2.71)	1.010		3, 79	0.393
Sex	20 (*f* = 10)	20 (*f* = 10)	20 (*f* = 10)	20 (*f* = 10)		0.000	3, 80	1.00
BMI	22.82 (2.36)	22.11 (2.00)	22.06 (2.79)	22.10 (2.46)	0.452		3, 79	0.717

We found no group differences for age (in years), sex, or Body Mass Index (BMI, in kg/m^2^).

Control Gain, Control group gain domain; Control Loss, Control group loss domain; Stress Gain, Stress group gain domain; Stress Loss, Stress group loss domain; f, female.

### Cortisol and sAA

**Figure 2:**
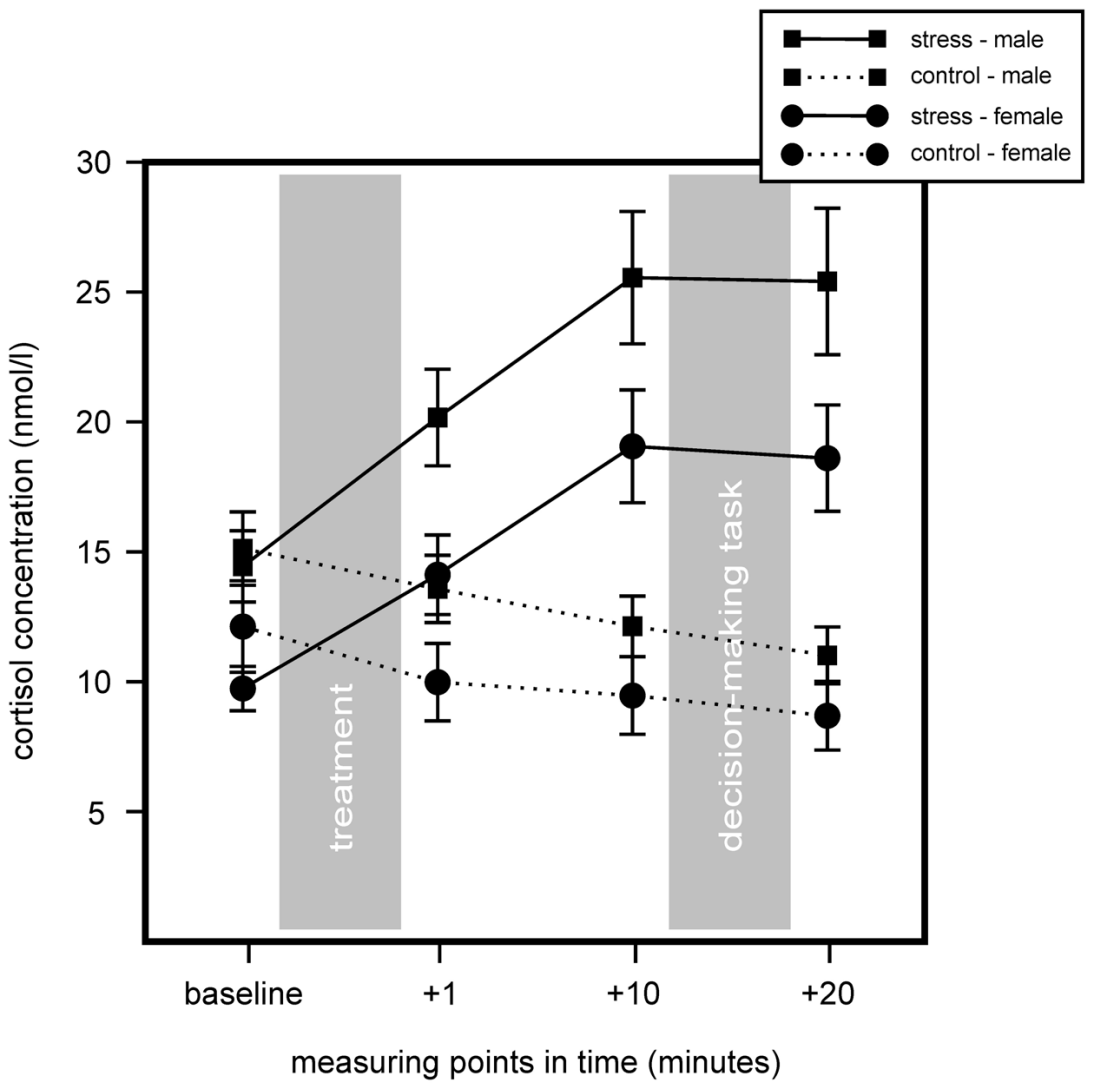
**Results of the salivary cortisol measures of the stress group (stress) compared to the control group (control) during the course of the experiment.** Stress increased cortisol within the stress group. Stressed males showed an overall higher cortisol concentration compared to females. Data represent means and standard errors.

We conducted a 2 (stress) × 2 (sex) × 4 (measurement time-points) repeated measurement ANOVA for cortisol and sAA to determine stress effects between stress and control groups. Results indicated an increase in cortisol for the stress group, but non for controls. Further, we found a sex difference with women showing overall lower cortisol concentrations. For cortisol significant main effects for stress, *F*_(1, 75)_ = 18.91, *p* < 0.001, η^2^ = 0.20, sex, *F*_(1, 75)_ = 7.72, *p* = 0.007, η^2^ = 0.09, and time, *F*_(1.50, 112.50)_ = 14.42, *p* < 0.001, η^2^ = 0.16, were found, as well as a significant interaction of Time × Stress, *F*_(1.50, 112.50)_ = 49.49, *p* < 0.001, η^2^ = 0.40. Follow up analyses with simple effects tests showed that stress and control group differed in sampling points 1, 10, and 20 min after stress cessation, all *F*_(1, 77)_ > 9.67, all *p* < 0.003, all η^2^ > 0.11. Males and females of the stress group differed in sampling points 1 min before and 1 min after the TSST, all *F*_(1, 38)_ > 6.31, all *p* < 0.016, all η^2^ > 0.14, and females showed a trend of lower cortisol in sampling points 10 and 20 min after stress cessation, all *F*_(1, 38)_ > 3.76, all *p* < 0.06, all η^2^ > 0.09. Results are illustrated in Figure 2.

**Figure 3:**
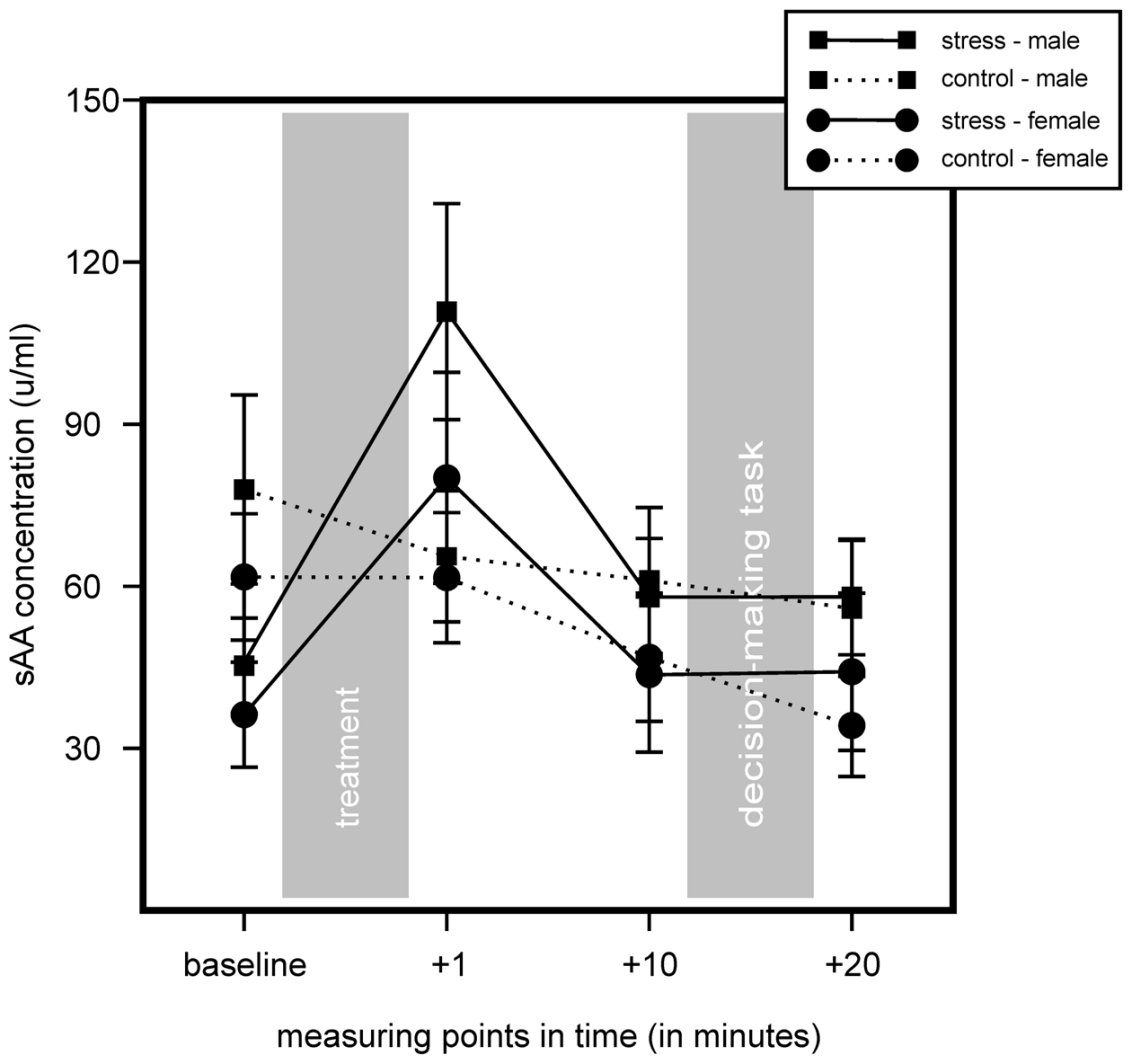
**Results of the salivary alpha-amylase (sAA) measures of stress group (stress) and control group (control) during the course of the experiment.** Stress increased sAA concentrations in the stress group, but not in controls. Data represent means and standard errors.

We found no increase of sAA concentration in controls and an increase in sAA in the stress group. We found no main effect for stress or sex, but a significant main effect for time, *F*_(2.32, 171.96)_ = 19.57, *p* < 0.001, η^2^ = 0.21, as well as a significant interaction of Time × Stress, *F*_(2.32, 171.96)_ = 14.37, *p* < 0.001, η^2^ = 0.16. Follow up simple effects tests showed a significant difference for baseline measurement, controls showing a higher sAA baseline, *F*_(1, 76)_ = 5.68, *p* = 0.02, η^2^ = 0.07. We also found a trend of an increase in the stress group 1 min after stress cessation, *F*_(1, 76)_ = 3.24, *p* = 0.076, η^2^ = 0.04. *T*-tests for independent samples showed a sAA increase (1 min after cessation of stress induction minus baseline) for the stress group, but not for controls, *t*_(69.11)_ = −5.65, *p* < 0.001. Results did not show any significant interactions with the factor sex. Results are illustrated in Figure 3.

### Positive and negative affect

We did not find any differences between groups or alterations over time for positive affect. Results showed an increase in negative affect for the stress group, but not controls. Sex differences were not found. We conducted a 2 (stress) × 2 (sex) × 2 (measurement time-points) ANOVA with repeated measurement for positive and negative affect, respectively. Results showed no significant main effect for group or time or any interactions with the factor stress for positive affect. For negative affect a significant main effect for time, *F*_(1, 76)_ = 32.10, *p* < 0.001, η^2^ = 0.30, and also a significant interaction of Time × Stress, *F*_(1, 76)_ = 24.11, *p* < 0.001, η^2^ = 0.24, could be shown. Descriptive statistics can be found in Table 2.

**Table 2:** **Results of the positive affect and negative affect schedule**.

	**Control *M* (*SD*)**	**Stress *M* (*SD*)**
PANAS-PAbase	29.43 (6.78)	30.75 (6.29)
PANAS-PAafter	30.25 (7.90)	30.15 (7.72)
PANAS-NAbase	11.68 (2.06)	12.95 (5.26)
PANAS-NAafter	12.08 (2.99)	18.55 (7.39)

Changes in positive and negative affect in the stress and control group are indicated.

Positive affect did not change over time and differences between the stress group (Stress) and the control group (Control) were not found. Negative affect increased over time in the stress group. Controls did not show altered negative affect (see results section). PANAS, Positive Affect Negative Affect Schedule; PA, positive affect; NA, negative affect; base, baseline measurement; after, measurement after stress cessation or resting, respectively.

### Decision-making performance

**Figure 4:**
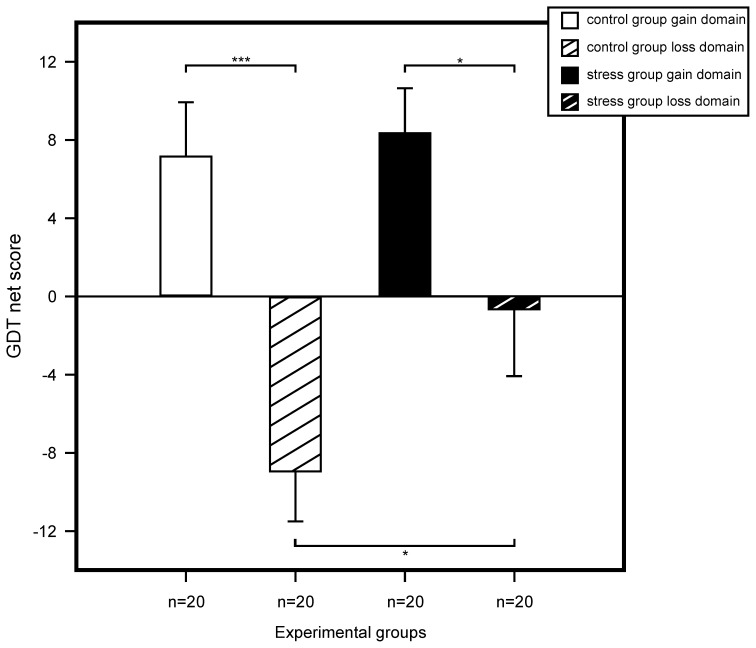
**Game of Dice Task (GDT) performance represented by the mean net score (number of less risky choices minus number of more risky choices).** Statistical analysis using an ANOVA revealed that among non-stressed participants riskier decisions were made in the loss domain. The same pattern was shown for stressed participants. Yet, for the loss domain, stressed participants showed less risky decision making compared to controls. We found no differences for controls and stressed participants of the gain domain. Data represent means over participants with standard errors. ^*^p < 0.05, ^***^p < 0.001.

We performed a multivariate ANOVA with GDT net score and reaction time as the dependent variables and with stress, domain, and sex as between subject factors. For GDT net score, results showed a trend for a main effect for stress, *F*_(1, 72)_ = 2.83, *p* = 0.097, η^2^ = 0.04, and a significant main effect for domain, *F*_(1, 72)_ = 19.42, *p* < 0.001, η^2^ = 0.21. We found no sex effect and none of the interactions of the three factors reached significance. Follow up analyses with simple effects tests showed significant riskier behavior for non-stressed participants within the loss compared to the gain domain, *F*_(1, 76)_ = 16.79, *p* < 0.001, η^2^ = 0.18. No difference was found in the gain domain between stressed and control participants, *F*_(1, 76)_ = 0.09, *p* = 0.759, η^2^ < 0.01. Most interestingly, in the loss domain we found a significant difference between controls and stressed participants, *F*_(1, 76)_ = 4.52, *p* = 0.037, η^2^ = 0.06, stressed participants making less risky decisions compared to controls. Results are illustrated in Figure 4.

**Figure 5:**
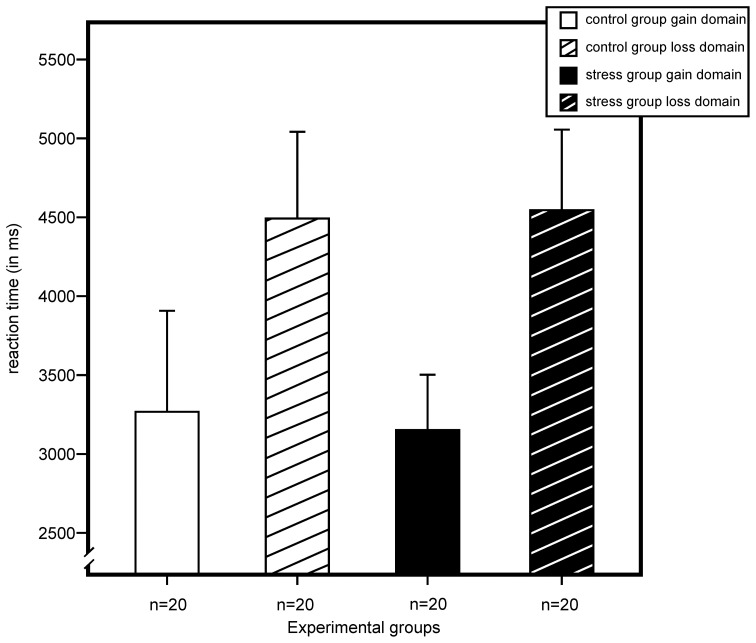
**Reaction time (in milliseconds) for gain and loss domain as measured by a modified version of the Game of Dice Task.** An ANOVA showed a main effect for domain with longer reaction times in the loss domains. Stress did not exert an effect on reaction time. Data represent means over participants with standard errors.

For reaction time, we found a significant main effect for domain, *F*_(1, 72)_ = 6.55, *p* = 0.013, η^2^ = 0.08, and no effects for stress, sex, or any of their interactions. As illustrated in Figure 5, results indicated longer reaction times for participants of the loss domain.

We analyzed the relationship between decision-making performance and cortisol concentration. Results were non-significant for linear, as well as for non-linear relationships.

## Discussion

In this study we investigated the effects of acute stress on framing with unequal distributed EVs across alternatives. After stress induction we found a rapid increase in SNS activity as indicated by sAA and the typical slower increase in cortisol concentrations indicating higher HPA axis activity in the stress group. The stress effect is supported by an increase in negative affect in the stress group after stress cessation.

Among non-stressed participants we found higher risk-taking in the loss compared to the gain domain despite better EVs associated with lower risk alternatives. This behavior is in accordance with studies investigating framed decision making [for a review see Rick (2011)] and may be explained by an urge to avoid losses by all means (Kahneman, 2003; Kahneman and Frederick, 2007). As such, these decisions are emotionally driven (Bechara and Damasio, 2005) and the applied decision-making strategy may have been the focus on lower losing probabilities only, instead of a calculative approach. This postulation is in line with results indicating participant's preference for alternatives with a low loss frequency (Lin et al., 2009; Aite et al., 2012; Koop and Johnson, 2012), thus choosing the better probability over the better economical outcome. Our results show no effect of higher EVs associated with less risky alternatives, as the framing effect remains stable and participants in the loss domain continue to make risky decisions. Consequently, our findings support the postulate that an emotional system is involved in framed decisions as suggested by dual-process theories (Druckman and McDermott, 2008; Cassotti et al., 2012) and are robust even after manipulation of the EV.

By modifying the GDT we could show that the framing effect is also present in more complex decision-making tasks. So far, most studies investigating framing effects in a monetary setting used simple lottery tasks (Tversky and Kahneman, 1986; Kuhberger, 1998; Porcelli and Delgado, 2009). Performing such a task, in a single trial participants are confronted with two alternatives only, each trial being self-contained. On the other hand, the GDT allows planning and strategizing by constantly choosing one out of altogether 14 always-present alternatives for a total of 18 trials. Thus, the stability of the emotional-driven framing effect seems also to suppress rationality in a task that is associated with higher cognitive functioning (Schiebener et al., 2011; Pabst et al., 2013b).

An additional finding is the increased response time in the loss compared to the gain domain. This may be due to a cost-benefit tradeoff associated with higher cognitive load in the loss compared to the gain domain (Gonzalez et al., 2005) and can be observed in the GDT as well.

Our central finding is the stress-reduced effect of loss framing, as we found less risky behavior of stressed compared to non-stressed participants. In the gain domain similar behavior of the stress and control group was shown. Earlier results of our groups indicate stress effects of riskier decision making in the original GDT that combines gains and losses (Starcke et al., 2008; Pabst et al., 2013a,b). The question is raised how these results may relate to one another and how the current results help to understand our earlier findings. First, we will discuss the stress effects on loss and gain domains followed by an integration of the results.

An explanation may be found in the influence of stress on the amygdala and the role of the amygdala in the framing effect. A patient study reported an elimination of loss aversion and less risky decisions in a loss domain in patients with amygdala lesions (de Martino et al., 2010). A PET study has shown that stress may lead to a decrease in amygdala activity (Pruessner et al., 2008) even though findings are somewhat compromised by the relatively poor spatial resolution of this imaging technique. Also, decreased amygdala activity has been found after cortisol administration in two fMRI fear conditioning studies (Merz et al., 2010, 2012). Moreover, studies reported that cortisol induced a loss of specificity in amygdala responding as well as a functional decoupling of the amygdala with the PFC (Henckens et al., 2010, 2012). We therefore hypothesize that stress may have decreased loss aversion due to altered amygdala activity. This could result in a diminished framing effect in loss domains (reduced fear of losing) after acute stress exposure. Clearly imaging studies investigating the relationship between stress, loss aversion, and amygdala activity are needed.

Moreover, studies show that other brain regions may also be involved in decisions in loss domains, specifically loss aversion, such as the striatum, the ventromedial PFC, the ventral anterior cingulate cortex, and the medial orbitofrontal cortex (Tom et al., 2007). Results indicate a relationship between activity decrease of these regions and increases in potential losses. Stress induced alterations of activity in these brain regions (Dedovic et al., 2009a) may be another potential explanation for less risky decision making in a loss domain.

Further, stress evokes a combination of negative emotions (Dickerson and Kemeny, 2004). Our results support this postulation, as there was an increase in negative affect after the TSST. Although findings on negative emotion and decision making have been somewhat inconsistent (Raghunathan and Pham, 1999; Lerner and Keltner, 2000), risk avoidance has been shown after induction of negative affective states (Yuen and Lee, 2003). In such a state participants may stronger evaluate higher losses and therefore tend to choose alternatives associated with lower losses, despite the higher loss frequency.

Another emotion-based explanation is the idea that negative emotions can be supportive of systematic processing (Schwarz, 1990) and are not necessarily a threat to rationality (Pfister and Bohm, 2008). Further support of this hypothesis is found in research indicating that individuals with a high ability of emotion regulation show reduced loss aversion due to a shift from an emotional to a rather cognitive strategy (Sokol-Hessner et al., 2009). Also, risk seeking behavior seems to stem from an overestimation of the emotional impact of a potential loss. Kermer et al. (2006) found that the actual emotional impact of a loss was smaller than the anticipated impact. If stress led to a rather cognitive strategy, stress may have decreased the effect of overestimating the emotional impact. Thus, it may be possible that participants actually applied a calculative strategy by making decisions based on a better EV.

In the literature it is postulated that high cortisol concentrations evoke a shift from higher to lower cognitive strategies (Porcelli and Delgado, 2009; Putman et al., 2010; Ossewaarde et al., 2011), such as from a goal-directed to a habitual strategy (Schwabe and Wolf, 2009; Schwabe et al., 2010a,b, 2011). May such a shift explain the less risky behavior of stressed participants in the loss domain? Two approaches may be applied. First, for non-stressed participants it is postulated that riskier decision making in a loss domain originates from an emotional approach, which is to avoid losses (Kahneman, 2003; Cassotti et al., 2012). It has been postulated that stress enforces habitual behavior and thus riskier behavior may be expected, as this would be the initial behavior [see Porcelli and Delgado (2009)]. The second approach is the potential advantage of bounded rationality and the use of heuristics. A rather large number of studies suggests that not higher cognitive processes, but intuitive and habitual responses may lead to better decision making (Gigerenzer, 2004; Brandstatter et al., 2006; Marewski et al., 2010). Instead of a calculative approach stressed participants of the loss domain may have laid focus on the monetary values, only. Thus, we believe that altered decision making may be explained by a strategy shift from higher to lower cognition. Yet, the underlying mechanisms need more investigation.

In the gain domain stress did not lead to riskier decision making as hypothesized. As decision making differs within gain and loss domains (Kahneman, 2003), we believe that a potential stress-induced reduction of amygdala activity (Pruessner et al., 2008; Merz et al., 2010) may not alter decision making in gain domains. This posit is supported by a positive relationship between amygdala inactivation and a reduction of preferences for larger, more costly rewards (Ghods-Sharifi et al., 2009).

Our study does not replicate the enhanced framing effect found by Porcelli and Delgado (2009), which may have two main reasons. First, the points in time of task performance were not similar. Porcelli and Delgado (2009) performed the decision-making task right after stress induction (which lasted only a few minutes). At their time of testing SNS activity, but most likely no cortisol increase occurred (Dickerson and Kemeny, 2004; Schwabe et al., 2008). In contrast we investigated the impact of stress-induced cortisol effects. Unfortunately, a comparison of cortisol concentrations during the decision tasks is not possible as Porcelli and Delgado (2009) measured skin conductance response, only. Decision making may depend on the temporal relation of task performance to the stressor and may evoke different behavior (Koot et al., 2013; Pabst et al., 2013a; Vinkers et al., 2013). Thus, Porcelli and Delgado (2009) may have found the enhanced framing effect under conditions of stress induced emotional arousal. Our findings in contrast may rather reflect the impact of stress induced cortisol elevations on decision making. Future pharmacological studies could address this issue more directly. Second, the simple lottery task and the more complex GDT are difficult to compare, as has already been discussed above. Also, we manipulated the EV, which could have led participants to apply a calculative strategy. Not so for Porcelli and Delgado (2009), where EVs did not differ.

How do the current results relate to the stress effects of riskier decisions in the more complex original GDT? The original GDT combines both gains and losses (Brand et al., 2005). Thus, riskier behavior is defined by choosing alternatives associated with possible high gains and low winning probabilities, but also high losing probabilities and an equivalent high loss. Our results in the stress loss domain show a decrease of risky decision making, as it may be evoked by a stress-induced reduction of amygdala activity (de Martino et al., 2010). Thus, we may hypothesize that under acute stress the focus in the original GDT is laid on gains rather than on losses or probabilities. This is in accordance with previous findings of striving for the big reward after cortisol administration even if it means to take a higher risk (Putman et al., 2010). If stress resulted in striving for the high reward, we should have found riskier decision making in the stress gain domain. Reasons for this missing stress effect have been discussed above.

We did not find any relationship between decision-making performance and cortisol concentration, similar to former studies of our groups (Pabst et al., 2013a,b). We believe that this might reflect indirect cortisol effects mediated by dopamine. As the release of dopamine seems to depend on the extent of cortisol secretion (Butts et al., 2011; Nagano-Saito et al., 2013), higher dopamine may have an impact on behavior (Floresco et al., 2008; Assadi et al., 2009; Mather and Lighthall, 2012). Further, studies have shown a high interindividual variance of GDT performance in stressed participants (Starcke et al., 2008), as well as in glucocorticoid sensitivity (Hellhammer et al., 2009; Kudielka et al., 2009), which may also account for a missing direct relationship. On a further note it should be mentioned that we did not take salivary samples beyond the time point 20 min after stress cessation and therefore are not able to provide information about negative feedback of the HPA axis. Future studies would benefit from the assessment of cortisol over a longer time span.

We did not observe any sex differences. Studies show that stress may lead to different behavior, as males may make more risky decisions and women may make less risky decisions (Lighthall et al., 2009, 2012). Also, men show slower learning of task contingencies with increasing cortisol. Performance of women seems to follow an inverted U-shaped function (van den Bos et al., 2009). Other results indicate poorer performance of both sexes at the beginning of task performance with woman showing an improvement later in the game, while men show continuously poor performance (Preston et al., 2007). Yet, for the GDT we observe neither sex differences nor sex by stress interactions (Starcke et al., 2008; Pabst et al., 2013b). Thus, the occurrence of sex differences might depend on the specific paradigms used.

In summary our results show the typical framing effect among non-stressed participants of riskier decision making in the loss compared to the gain domain. Stress did not amplify the framing effect but instead reduced the amount of risky decisions taken in the loss domain. Thus, the combination of loss expectancies and stress interacted in a way which reduced risk-taking behavior. Underlying psychological and neurobiological mechanisms are in need of further explorations.

### Conflict of interest statement

The authors declare that the research was conducted in the absence of any commercial or financial relationships that could be construed as a potential conflict of interest.
